# Diagnostic and prognostic value of the gasdermins in gastric cancer

**DOI:** 10.1590/1414-431X2024e13817

**Published:** 2024-11-25

**Authors:** Yeqiong Xu, Jie Chen, Ping Wang, Huanhuan Chen, Yilin Zhao, Xuexian Cao, Chuandan Wan, Yulan Gu

**Affiliations:** 1 Center Laboratory of Changshu Medical Examination Institute, Changshu, Jiangsu Province, China; 2 School of Preclinical Medicine, Wannan Medical College, Wuhu, China; 3 Department of Oncology, Affiliated Changshu Hospital of Nantong University, Suzhou, China

**Keywords:** Gasdermin, Pyroptosis, Gastric cancer, Bioinformatics, Diagnosis, Prognosis

## Abstract

Pyroptosis has attracted attention due to its role in various cancers. Recently, gasdermins (GSDMs) involved in pyroptosis have been reported to be associated with several types of cancers. However, the role of GSDMs expression in the diagnosis and prognosis of gastric cancer (GC) is still not well understood. We analyzed the transcriptional and prognostic information and the role of GSDMs in patients with GC from TIMER, UALCAN, Human Protein Atlas (HPA), GEPIA, and Kaplan-Meier Plotter databases. The cBioPortal platform was used to discover the genetic alterations, significance, and networks of GSDMs. Furthermore, STRING, Cytoscape, and TIMER were used to explore functional enrichment and immunomodulation. GSDMB, GSDMC, GSDMD, and GSDME were more highly expressed in GC than in normal tissues in the TIMER database. Moreover, survival analyses in two databases showed that high expression of GSDME was related to shorter overall survival (OS) in patients with GC. Additionally, functional enrichment revealed that GSDMs may be involved in endopeptidase activity, peptidase regulatory activity, and cysteine peptidase activity. GSDMs correlated with infiltration levels of immune cells in GC, and GSDME correlated with the infiltrating level of CD4+ T, CD8+ T, neutrophils, macrophages, and dendritic cells. This study indicated the potential diagnostic and prognostic value of GSDMs in GC. Our results showed that GSDME could play a significant oncogenic role in GC diagnosis and prognosis. However, our bioinformatics analyses should be validated in further prospective studies.

## Introduction

Gastric cancer (GC), one of the most prevalently malignant tumors in East Asia, affects both the physical and mental health of patients. It has been reported that an estimated 968,350 new GC cases and 659,853 GC related deaths will occur worldwide in 2022, making GC fifth in both cancer-specific incidence and mortality (1). In spite of multiple therapy strategies, including chemotherapy, radiotherapy, immunotherapy, and surgical resection, which have made significant progress recently, the cure rate of GC remains suboptimal, and the five-year survival rate for patients with GC remains unfavorable due to local relapse or distant metastasis (2), causing a major burden on families and society. Therefore, new biomarkers for molecular pathology diagnosis and prognosis prediction are urgently needed to effectively improve prognosis and individualized treatment.

Pyroptosis is a proinflammatory process which features plasma membrane rupture due to gasdermin pore formation (3-6). Gasdermins (GSDMs), which are critical for pyroptosis induction (5), consist of a family of pore-forming proteins, GSDMA, GSDMB, GSDMC, GSDMD, GSDME (DFNA5), and PJVK (DFNB59), which separately display differential tissue expression (6). Recently, GSDMs have been reported to be involved in a diversity of cellular activities, such as inflammation, cell proliferation, differentiation, and cell death (7,8), suggesting that GSDMs are associated to various oncological pathologies, such as GC (9,10), breast cancer (11), colorectal cancer (12), and lung cancer (13). Although GSDMs have been related to GC, their specific role in tumorigenesis and tumor progression remains unknown. Therefore, an in-depth investigation of the association between GSDMs and GC will provide new directions and targets for the detection, treatment, and prevention of GC.

The present study was carried out to investigate the function and molecular mechanism of GSDMs in GC biology.

## Material and Methods

### TIMER database analysis

TIMER 2.0 (http://timer.comp-genomics.org/) was used to analyze the expression levels of GSDMs in various normal and tumor cells and in immune cells infiltrating thirty-one tumor types (14). The scatter plots of GSDMs were constructed to display the purity-corrected partial Spearman's rho values and statistical significance. The results showed that a positive value of expected genes indicated high expression in the tumor cells, while a negative value indicated overexpression in the microenvironment. In the current study, we analyzed the GSDMs expression level in 6 tumor-infiltrating immune cells (including B cells, CD4+ T cells, CD8+ T cells, neutrophils, dendritic cells, and macrophages).

### UALCAN database

UALCAN database is an online database publicly available. It was used to evaluate the mRNA levels of GSDMs in GC according to OMICS (including TCGA, MET500, and CPTAC platforms) (15). Furthermore, this database was also used to analyze the mRNA expression of GSDMs in GC stage and grade subgroups.

### GEPIA database

GEPIA is a publicly accessible tool for estimating the prognosis of GC patients according to GSDMs expressions based on the GTEx and TCGA databases (16), with almost ten thousand tumor samples.

### Kaplan-Meier plotter

Kaplan-Meier plotter is used to analyze the association between different gene expressions and survival in several tumors. Herein, the database was used to explore the prognosis of GC patients based on GSDMs expressions.

### HPA database analysis

HPA serves as a database of representative protein levels of nearly twenty highly common tumors by immunohistochemistry (17). This database was used to study the levels of GSDMs expression in GC and corresponding normal tissues.

### cBioPortal database analysis

The cBioPortal database (https://www.cbioportal.org/results/oncoprint?data_priority=0&tab_index=tab_visualize&Action=Submit&session_id=607bb3d9e4b015b63e9e6853) was used to analyze gene alterations, copy number alterations, mRNA expression Z scores, and protein expression Z scores.

### STRING and Cytoscape

STRING is an online tool for the analysis of protein-protein interactions (PPI) (18). STRING provides the prediction index including protein/gene interactions, co-expression, protein domains, subcellular localization, and signaling networks. Furthermore, Cytoscape is as a public platform for network analysis and visualization. Using these platforms, we further analyzed the proteins interacting with members of GSDMs family.

### Statistical analysis

Statistical analysis of the data was conducted in the R environment (version 3.6.3). All statistical tests were bilateral, and a P-value <0.05 was considered statistically significant.

## Results

### Transcriptional levels of GSDMs in distinct types of tumors

**Figure 1:**
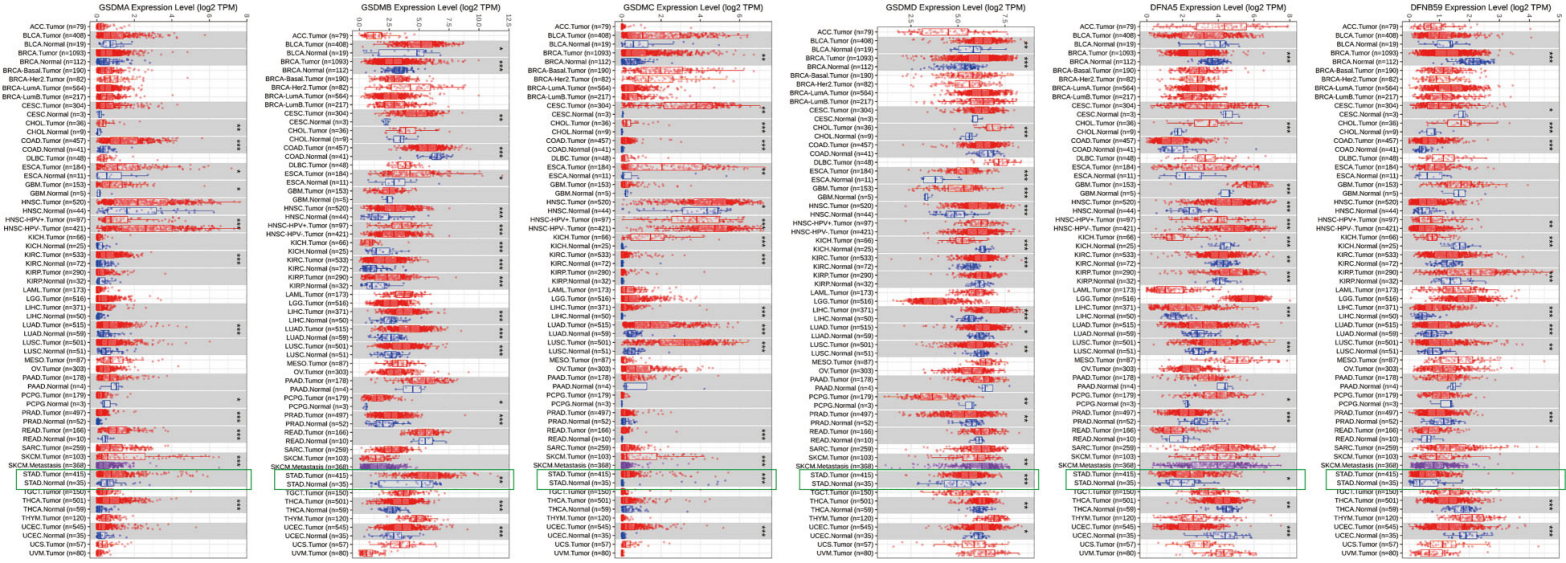
Gasdermins (GSDMs) levels among multiple tumors and normal cells via TIMER analysis. *P<0.05, **P<0.01, ***P<0.001; unpaired *t*-test.

To estimate the critical effect of GSDMs involved in multiple carcinogenesis, transcriptional expression levels of six GSDMs in tumors and counterpart normal tissues were compared by TIMER database (Figure 1). Our results indicated that GSDMs were highly expressed in numerous tumors, including cholangiocarcinoma, uterine corpus endometrial carcinoma, bladder urothelial carcinoma, and GC. Among all types of tumors, GSDMB, GSDMC, GSDMD, and GSDME had higher expression than normal tissues.

### Association between expression of GSDMs and clinicopathological parameters of patients with GC

**Figure 2:**
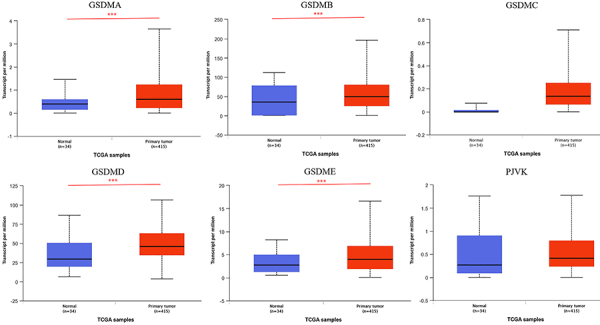
Gasdermins (GSDMs) expression levels in gastric cancer and normal tissue in UALCAN database. Data are reported as medians and interquartile range. ***P<0.001; Wilcoxon test.

**Figure 3:**
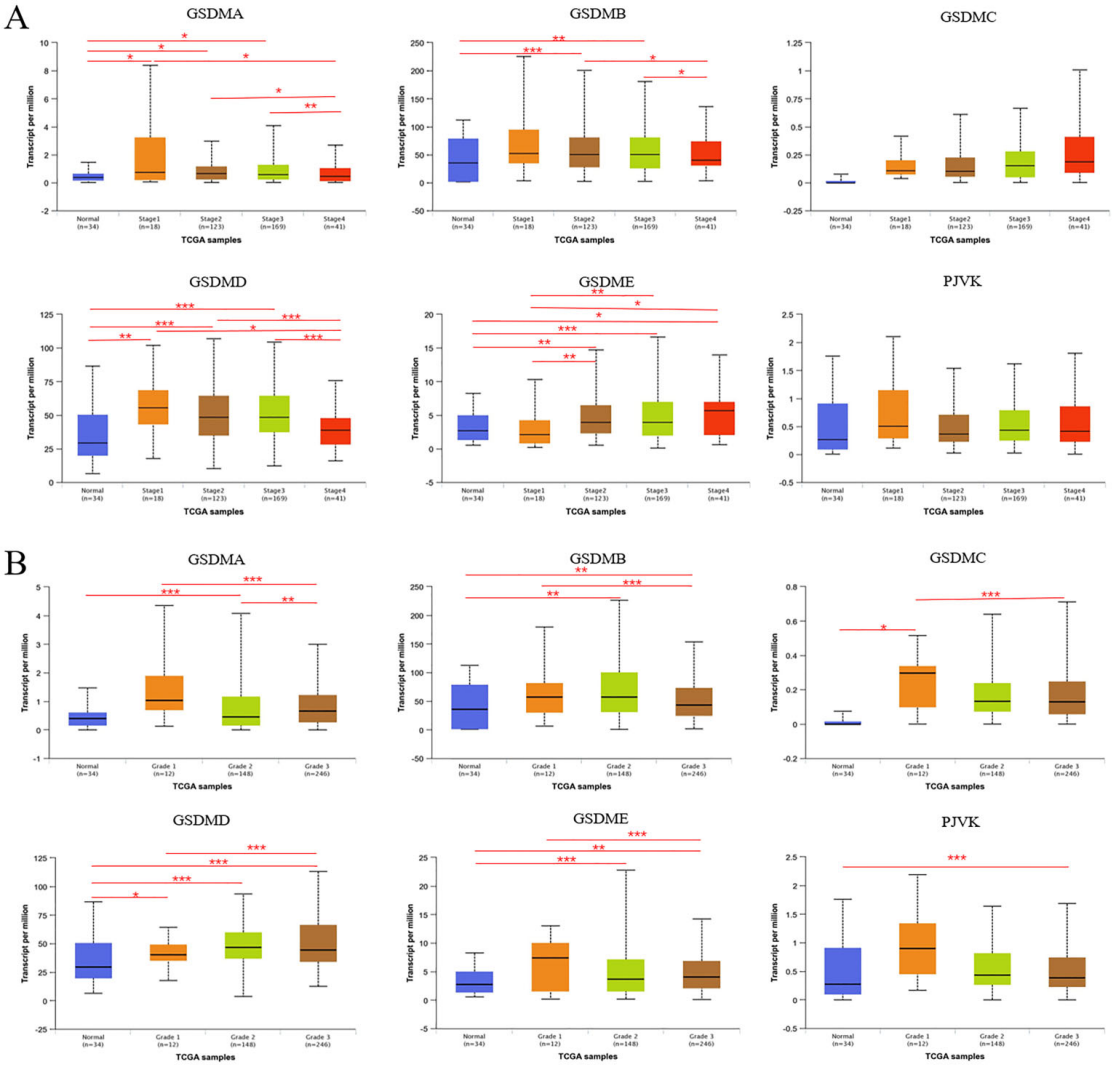
Gasdermins (GSDMs) mRNA levels in gastric cancer (GC) patients with different clinicopathological parameters in the UALCAN platform. Association between GSDMs mRNA expression and different GC tumor stages (**A**) and grades (**B**). Data are reported as medians and interquartile range. *P<0.05, **P<0.01, ***P<0.001; Kruskal-Wallis test.

The mRNA expression levels of GSDMs in GC and normal tissues were compared by UALCAN database. The results showed that the levels of GSDMA, GSDMB, GSDMD, and GSDME were higher in GC than in normal tissues (Figure 2). Two key clinicopathological parameters of GC, tumor stage and grade, were further analyzed according to the expression of GSDMs. GSDMA, GSDMB, GSDMD, and GSDME displayed significantly higher expression in tumor stage 2 and 3 than in normal tissues. Compared with normal tissues, GSDMA and GSDMD showed higher expression levels in tumor stage 1. The expression levels of all GSDMs (except GSDME) had no difference between tumor stage 4 and normal tissues (Figure 3A). In terms of tumor grade, the expression level of GSDMD gradually increased from tumor grade 1 to 4 and all were higher than in normal tissues. The expression levels of GSDMB and GSDME were higher in tumor grades 2 and 3 compared to normal tissues, whereas GSDMA expression level was significantly increased in tumor grade 2 compared to normal tissues. GSDMC and PJVK expression levels in tumor grade 1 and 3 differed from normal tissues, respectively (Figure 3B).

**Figure 4:**
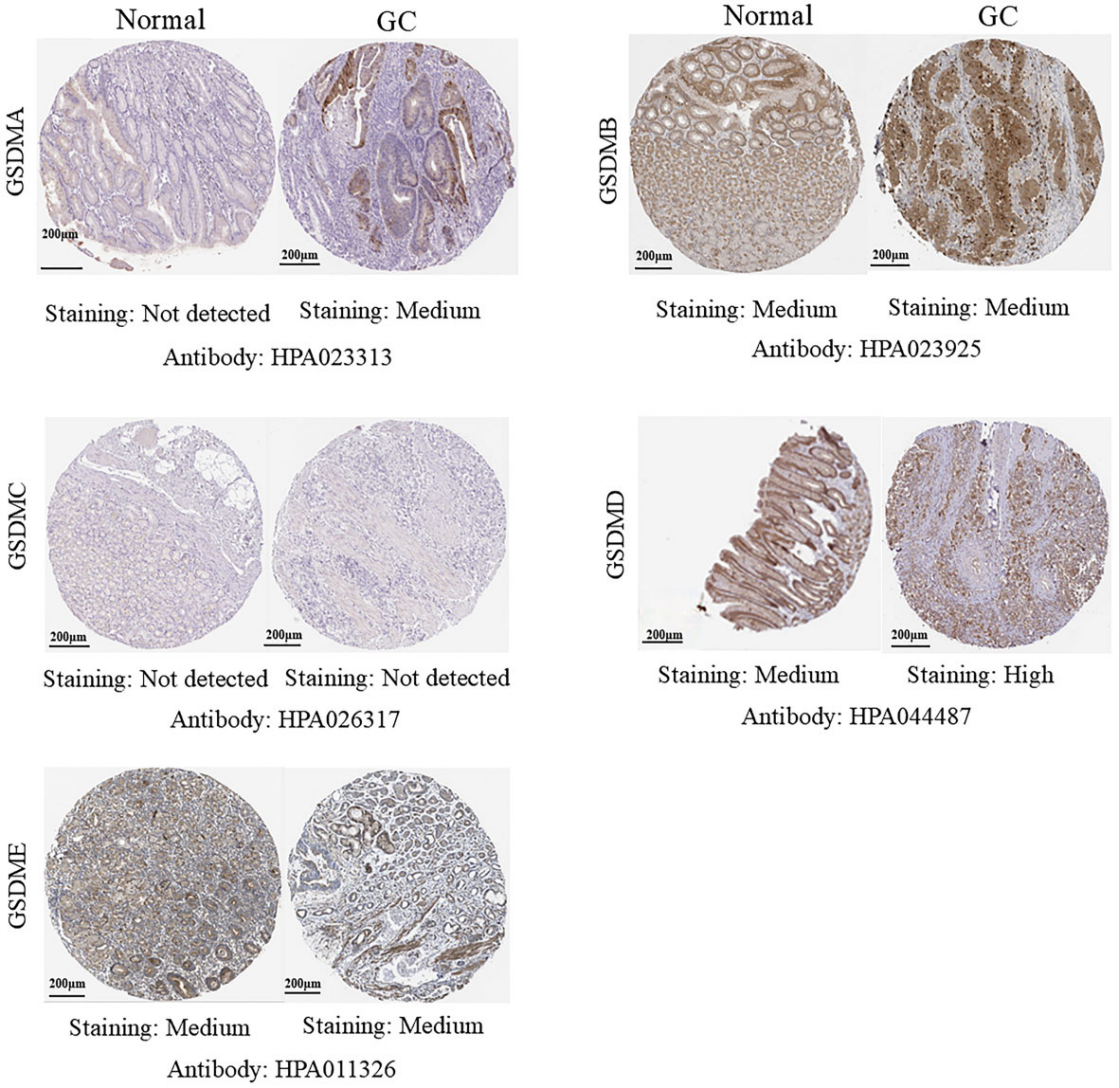
Gasdermins (GSDMs) expression in patients with gastric cancer (GC) using immunohistochemistry (HPA). Scale bar: 200 μm.

The results of immunohistochemistry indicated that GSDMA and GSDMD proteins were overexpressed in the GC tissues compared to normal tissues, which was in line with the findings of mRNA expression levels (Figure 4). However, GSDMB, GSDMC, and GSDME proteins showed no difference between GC tissues and the normal counterparts, and PJVK did not have immunohistochemistry data.

### Correlation between mRNA expression levels of GSDMs and prognosis in GC patients

**Figure 5:**
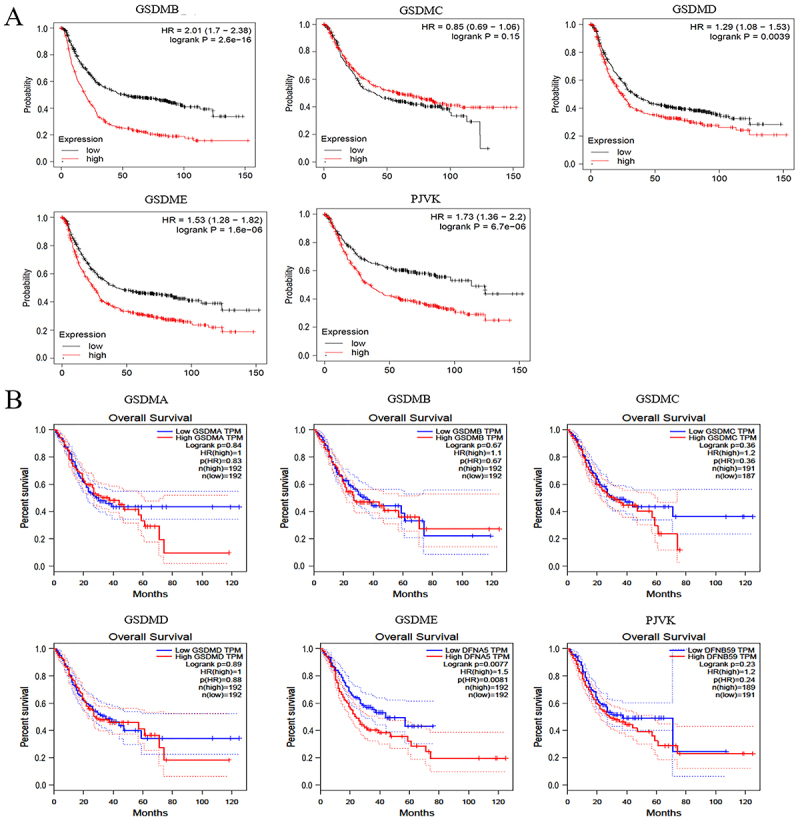
Prognostic value of gasdermins (GSDMs) in gastric cancer (GC) patients. **A**, Correlation between GSDMs mRNA levels and overall survival by the Kaplan-Meier plotter database. **B**, Correlation between GSDMs mRNA levels and GC patients overall survival via the GEPIA database.

Furthermore, the key role of GSDMs in overall survival (OS) of GC patients was explored. The results of Kaplan-Meier plotter and GEPIA appeared inconsistent. The former showed that high expression levels of GSDMB, GSDMD, GSDME, and PJVK were correlated with worse OS in GC patients (Figure 5A). However, no data of GSDMA was retrieved from the above database. The latter revealed that overexpression level of GSDME was associated with shorter OS in GC patients (Figure 5B).

### Predicted pathways and functions of changing in GSDMs and the frequently varied neighbor genes in GC patients

**Figure 6:**
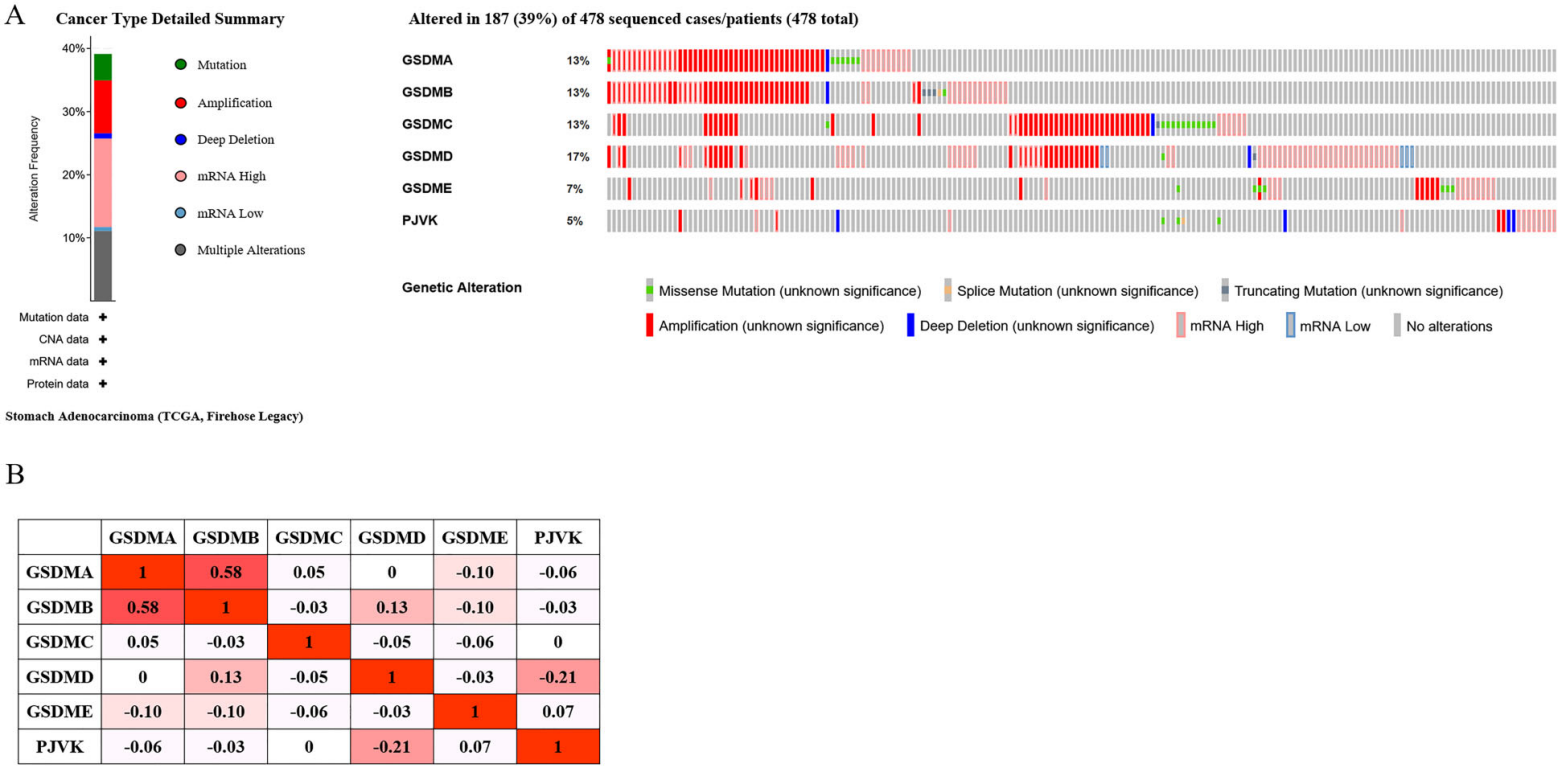
Gasdermins (GSDMs) expression and genetic mutation analysis in gastric cancer (GC) using cBioPortal database. **A**, GSDMs levels and genetic mutations in GC. **B**, Correction analysis between different GSDMs in GC.

Alterations of GSDMs were detected in 187 samples (39%). The alteration frequency of GSDMA, GSDMB, GSDMC, GSDMD, GSDME, and PJVK were 13, 13, 13, 17, 7, and 5% based on cBioPortal database (Figure 6A). The main genetic alteration types of GSDMs were amplification and high mRNA expression. However, missense mutation, splice mutation, truncating mutation, and deep deletion were rare in GSDMs. Meanwhile, the homologous correlations of GSDMs were analyzed, showing a strong correlation in GSDMA and GSDMB (R=0.58), and weak correlation in PJVK and GSDMD (R=-0.21) (Figure 6B).

**Figure 7:**
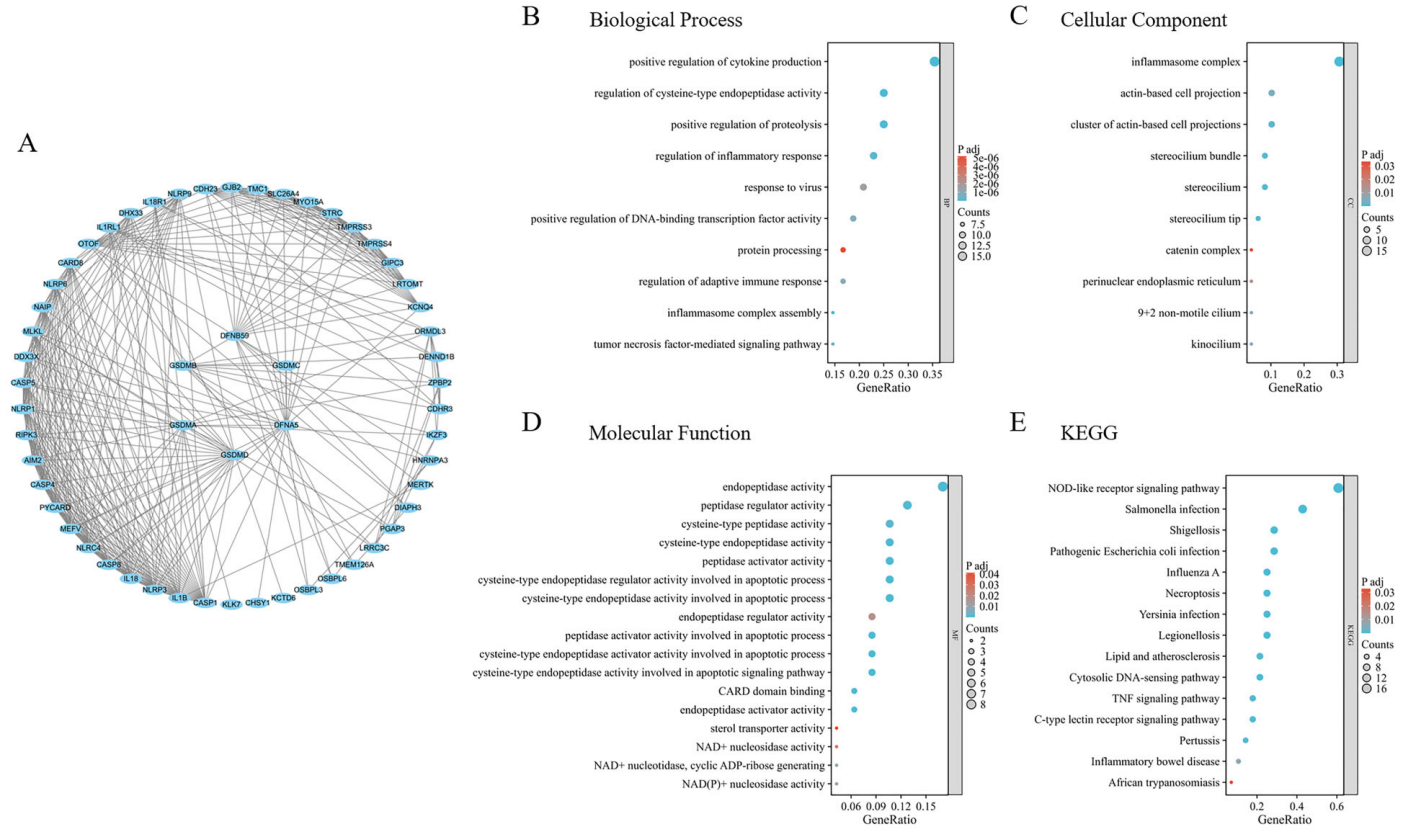
Function enrichment and pathways of gasdermins (GSDMs) and co-expression genes in gastric cancer (GC). **A**, Network for GSDMs and the most frequently altered co-expressed genes in GC using STRING and visualization by Cytoscope. **B**, Biological process analysis for GSDMs-associated co-expressed genes. **C**, Cellular component analysis of GSDMs associated co-expressed genes. **D**, Molecular function analysis of GSDMs-associated co-expressed genes. **E**, KEGG pathway analysis of GSDMs-associated co-expressed genes.

Moreover, STRING and Cytoscape were applied to uncover the relevant co-expression of genes and their association with GSDMs, showing that interactions among six genes were significant in this network (Figure 7A). Furthermore, Gene Onotology (GO) annotation and Kyoto Encyclopedia of Genes and Genomes (KEGG) pathway analyses were used to determine their biological function. The top-ranking biological processes regarding GSDMs were cytokine production, regulation of cysteine-type endopeptidase activity, regulation of proteolysis, regulation of inflammatory response, and response to virus (shown in Figure 7B). As for cellular components of GSDMs, the results focused on inflammasome complex, actin-based cell projection, cluster of actin-based cell projections, stereocilium bundle, and stereocilium (Figure 7C). Moreover, the highly enriched molecular functions of GSDMs were endopeptidase activity, peptidase regulator activity, cysteine-type peptidase activity, cysteine-type endopeptidase activity involved in apoptotic process, and peptidase activator activity (Figure 7D). The KEGG pathway analysis indicated that the top five genes were involved in the NOD-like receptor signaling pathway: Salmonella infection, Shigellosis, Pathogenic Escherichia coli infection, and Influenza A (Figure 7E).

### Correlation between mRNA expression levels of GSDMs and immune cell infiltration in GC patients

**Figure 8:**
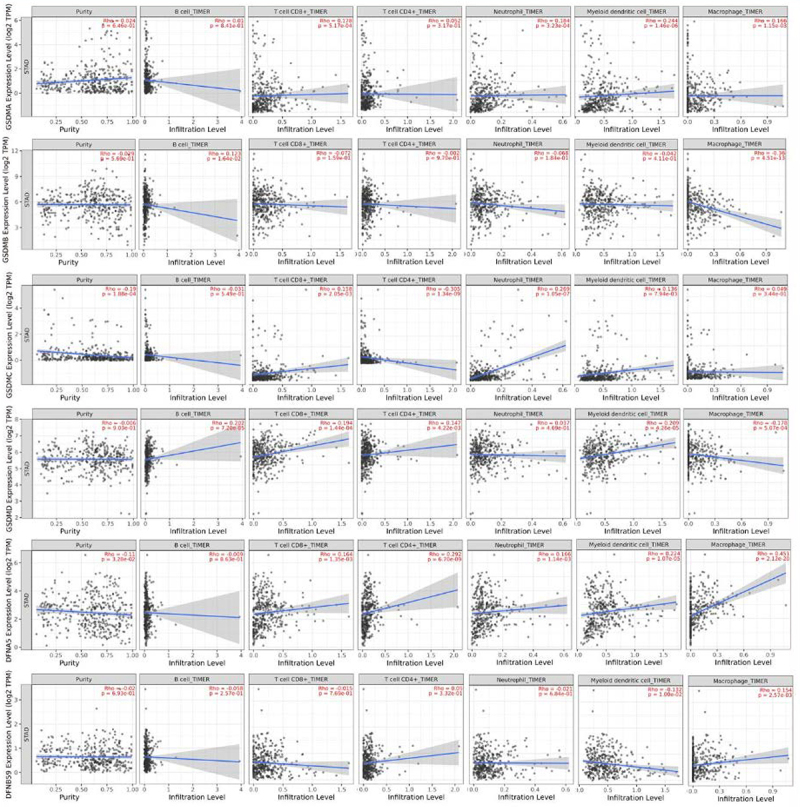
Gasdermins (GSDMs) correlating with infiltration levels of immune cells in gastric cancer via TIMER platform.

TIMER 2.0 was utilized to correlate the expression levels of GSDMs with immune cell infiltration in GC (Figure 8). mRNA expression levels of GSDMC and GSDME were correlated to tumor purity. More specifically, GSDMA was positively correlated with CD8+ T cell (Rho=0.178, P=5.17e-04), neutrophil (Rho=0.184, P=3.23e-04), dendritic cell (Rho=0.244, P=1.46e-06), and macrophage (Rho=0.166, P=1.15e-03) infiltration. Moreover, GSDMB was positively correlated with B cell (Rho=0.123, P=1.64e-02) infiltration, while negatively correlated with macrophage (Rho=-0.360, P=4.51e-13) infiltration. Similarly, a positive correlation was observed between GSDMC and CD8+ T cell (Rho=0.158, P=2.05e-03), dendritic cell (Rho=0.136, P=7.94e-03), and neutrophil (Rho=0.269, P=1.05e-07) infiltration, while there was a negative correlation with CD4+ T cell (Rho=-0.305, P=1.34e-09) infiltration. Likewise, a positive correlation was observed between B cell (Rho=-0.202, P=1.34e-09), CD4+ T cell (Rho=0.158, P=2.05e-03), and dendritic cell (Rho=0.136, P=7.94e-03) infiltration and GSDMD expression level. Conversely, a negative correlation was found between GSDMD expression level and macrophage (Rho=-0.178, P=5.07e-04) infiltration. However, CD4+ T cell (Rho=0.292, P=6.70e-09), CD8+ T cell (Rho=0.164, P=1.35e-03), neutrophil (Rho=0.166, P=1.14e-03), dendritic cell (Rho=0.224, P=1.07e-05), and macrophage (Rho=0.451, P=2.12e-20) infiltration was positively correlated with GSDME expression level. Finally, PJVK positively correlated with macrophage (Rho=0.154, P=2.57e-03) infiltration, while a negative correlation with dendritic cell (Rho=-0.132, P=1.00e-02) infiltration was found.

## Discussion

Today, GC still represents a major health burden globally due to metastasis and high tumor-related death. While early diagnosis is important for prolonging survival time of GC, it is still full of challenges. Therefore, exploring novel predictive markers for early detection, progression, and prognosis of GC is urgently needed. So far, a great deal of evidence has demonstrated that GSDMs dysregulation can influence the development of tumors (11-13), but further analyses in GC have yet to be conducted.

In our current work, RNA expression and prognostic values of GSDMs in GC were explored using public databases. In TIMER database, GSDMB, GSDMC, GSDMD, and GSDME were overexpressed in GC compared to normal tissues, while in UALCAN database, GSDMA, GSDMB, GSDMD, and GSDME were higher in GC compared with normal tissues. GSDMB, GSDMD, and GSDME showed highly consistent trends, indicating a potential role in tumorigenesis. Furthermore, the correlation between tumor stage and grade and expression of GSDMs was further analyzed. The results suggested that advanced tumor stage might have distinct mechanisms blocking the expression of GSDMs. From the protein expression level, GSDMA and GSDMD showed consistent results, being highly expressed in GC. The trend suggested that GSDMs played a role in oncogenesis and tumor progression. In addition, the GSDME had a similar result, showing its prognostic value in GC. Many studies have reported that GSDME is involved in oncogenesis and chemoresistance. GSDME knockdown could markedly suppress the growth of hepatocellular carcinoma (19). GSDME-EGFR interaction was involved in the development of non-small cell lung cancer (20), which could open the horizon of cancer pathogenesis. By inducing caspase 3-GSDME pathway, CC-115 exerted antitumor effects in lung adenocarcinoma (21). It is believed that GSDME could be a hopeful predictive and therapeutic marker of multiple tumors.

To further elucidate the underlying mechanism of GSDMs in GC tumorigenesis, progression and prognosis, the biological function and immune infiltration related to GSDMs were investigated. The molecular functions regarding GSDMs were focused on protease activity. It is reported that proteolytic networks could regulate tumor angiogenesis, invasion, and signaling pathways in the tumor microenvironment (TME) involving chemokines, cytokines, and kinases (22). Emerging evidence has indicated that the immune microenvironment plays a key role in GC tumorigenesis, progression, and prognosis (23-26) and has become a new determinant of immunotherapy response and clinical outcome (27,28).

Tumor infiltrating lymphocytes (TILs) (including T cells, B cells, and NK cells) were increased in GC, especially in advanced cases, which suggests that TILs may be associated with tumor immune escape and dysfunction of T cells in GC (29). Moreover, several types of GC showed immune tolerance and were infiltrated with high levels of TILs and low PD-L1 (29). In our study, the expression level of GSDME had a positive correlation with CD4+ T cell, CD8+ T cell, neutrophil, dendritic cell, and macrophage infiltration, suggesting that GSDME may be involved in the immunomodulatory mechanisms of GC. Recently, it was recognized that the TME has a role in tumor proliferation and metastasis, and single-cell RNA sequencing revealed that the TME of GC was filled with immune cells including macrophages, dendritic cells, and Tregs (30). Oshi et al. (31) reported that GC with high angiogenesis score was significantly associated with a lower infiltration of Th1, Th2, and dendritic cells, and a higher infiltration of M1 macrophages, and was also associated with shorter survival.

To the best of our knowledge, tumor-associated macrophages (TAMs), a critical member of TME, participated in the tumorigenesis and development of GC (32,33). Recently, novel studies have indicated that TAMs are involved in tumor progression via taking part in immunomodulation of GC (34-36). Huo et al. (37) demonstrated the prognostic value of TAMs in GC and pointed out a higher macrophage infiltration in TAM of GC patients together with a worse prognosis. It has been reported that elevated levels of peripheral or intratumoral neutrophils in GC patients were accompanied with poor survival (38,39), which indicated that neutrophils play an important role in promoting the pathological process of GC. A recent study reported pathogenic roles of neutrophils in GC through a novel mechanism: tumor tissue can attract neutrophils by CXCL6/CXCL8‐CXCR1 interactions and lead to the accumulation of neutrophils in GC (40). There have been different opinions about the role of tumor-infiltrating immune cells recently. It is well established that immune cells are a double-blade sword, potentially promoting and inhibiting tumor development, which needs further investigation. The present study indicated that there are positive relationships between GSDME expression level and infiltration of immune cells, suggesting that GSDME might be critical in the regulation of immune cell infiltration in GC.
